# Supplementary material for: Monocyte Subsets and Serum Inflammatory and Bone-Associated Markers in Monoclonal Gammopathy of Undetermined Significance and Multiple Myeloma
Source: Cancers (Basel). 2021 Mar 22;13(6):1454. doi: 10.3390/cancers13061454 (PMC8004952; doi:10.3390/cancers13061454)
Supplement: Supplementary file 1 [file cancers-13-01454-s001.pdf]

Supplementary Materials: Monocyte Subsets and Serum Inflammatory and Bone-Associated Markers in Monoclonal Gammopathy of Undetermined Significance and Multiple Myeloma

Daniela Damasceno, Julia Almeida, Cristina Teodosio, Luzalba Sanoja-Flores, Andrea Mayado, Alba Pérez-Pons, Noemi Puig, Paula Arana, Bruno Paiva, Fernando Solano, Alfonso Romero, Sergio Matarraz, Wouter B.L. van den Bossche, Juan Flores-Montero, Brian Durie, Jacques J.M. van Dongen and Alberto Orfao on behalf of the TiMaScan Study Group

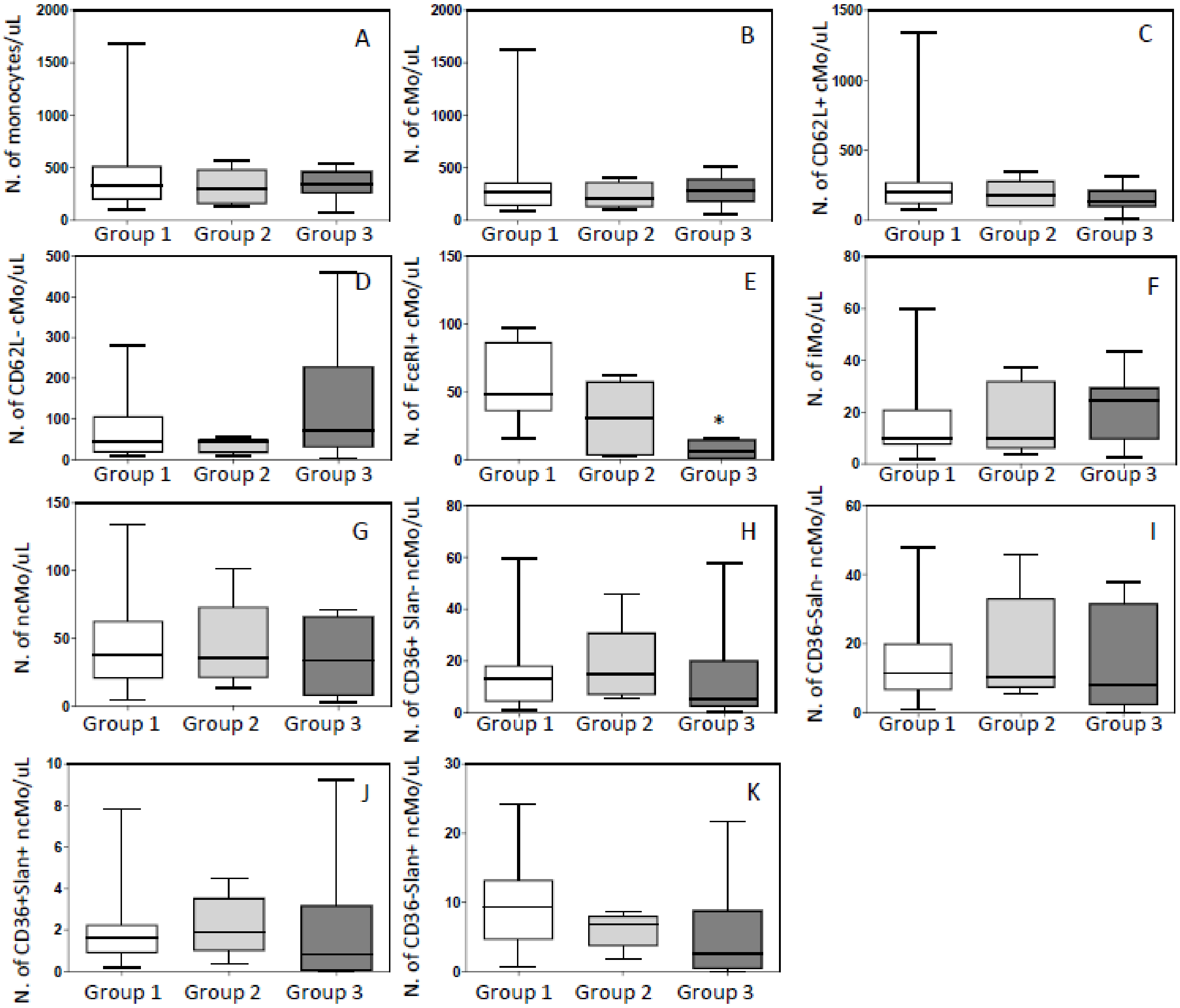

Figure S1. Distribution of total monocytes (panel A) and monocyte subsets (panels B-K) in blood of 4 MGUS, 5 SMM, 16 MM and 5 HD grouped by TSNE (groups 1, 2 and 3). Notched boxes extend from the 25th to the 75th percentile values; the lines in the middle and vertical lines correspond to median values and the 5th and 95th percentiles, respectively. Statistical significant differences ($p<0.05$) were observed vs *Group 1. cMo: classical monocytes; iMo: intermediate monocytes; ncMo: non-classical monocytes.

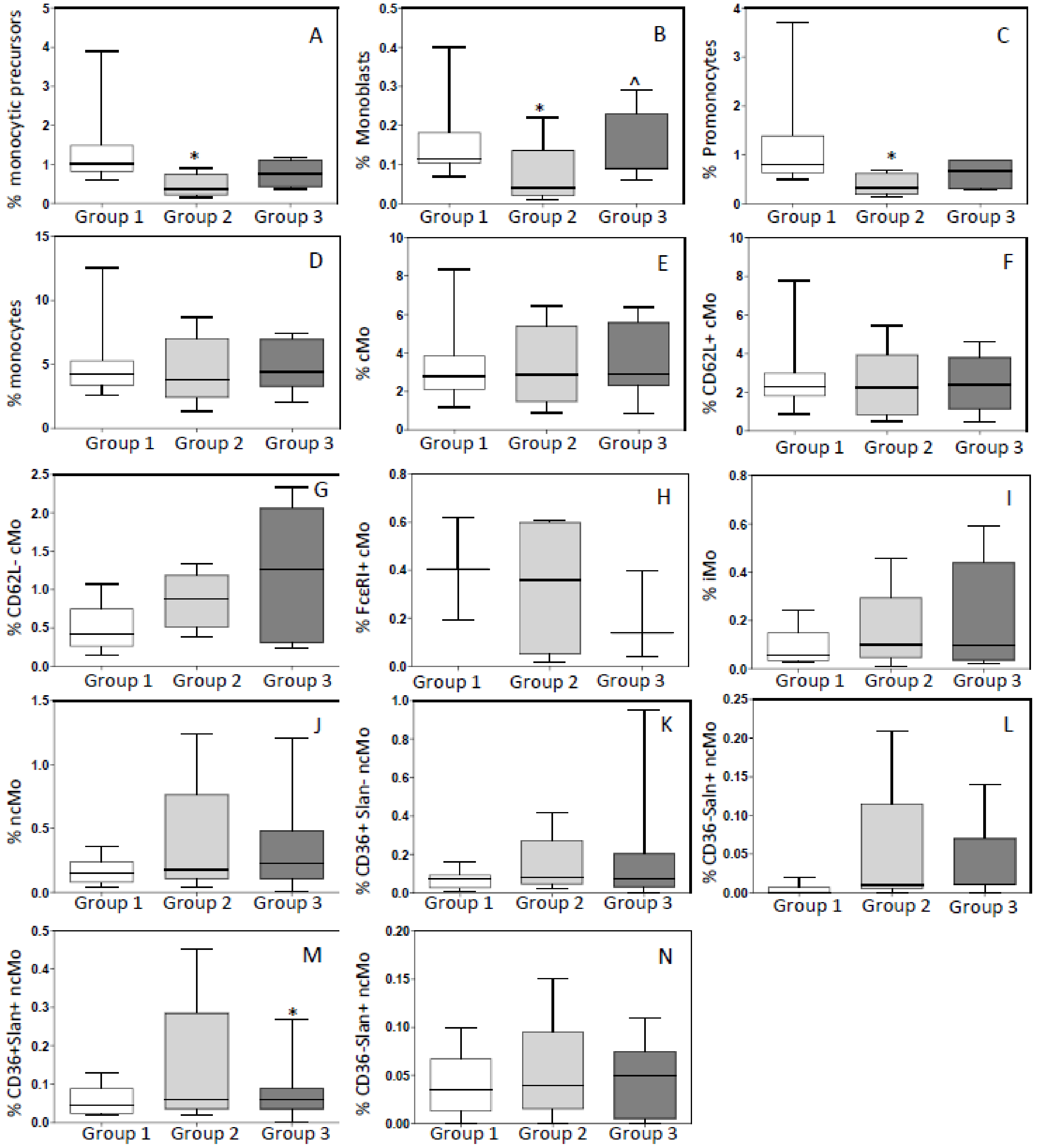

Figure S2. Distribution of monocytic precursors (total precursors, monoblasts and promonocytes) (panels A-C), total monocytes (panel D) and their subsets (panels E-N) in bone marrow of 4 MGUS, 5 SMM, 16 MM and 5 HD grouped by T-SNE (groups 1, 2 and 3). Notched boxes extend from the 25th to the 75th percentile values; the lines in the middle and vertical lines correspond to median values and the 5th and 95th percentiles, respectively. Statistical significant differences ($p<0.05$) were observed vs *Group 1 and ^Group 2. cMo: classical monocytes; iMo: intermediate monocytes; ncMo: non-classical monocytes.

Table S1. Clinical characteristics of MGUS, SMM and MM patients and HD.

Patient Characteristics	HD		MGUS		SMM		MM	
	PB	BM	PB	BM	PB	BM	PB	BM
Number of cases	97	15	22	19	13	13	88	81
Age, years, median (range)	62 (32-92)	59 (31-83)	67 (31-85)	70 (44-85)	64 (43-86)	63 (43-86)	72 (45-85)	71 (45-85)
Sex, males/females, (%)	50/50	66/34	54/46	58/42	38/62	54/46	47/53	42/58
MM ISS stage, I/II/III, (%)							37/23/40	
MM R-ISS stage, I/II/III, (%)							25/54/21	
Mayo Clinic risk score, 0/1/2/3, (%)			26/26/42/6		23/61/16/0			
Osteolytic lesions, No/Yes, (%)			100/0		100/0		44/56	
Leukocytes (WBC), cells/µl, median (range)	8310 (6120-10390)		6582 (4078-10147)		5885 (5346-6483)		5641 (2721-10907)	
Hemoglobin, g/dL, median (range)	NA		15 (12-18)		13 (12-16)		11 (6-17)	
Serum calcium, mg/dL, median (range)	NA		9.4 (8.7-9.8)		9.4 (8.7-10)		9.3 (7.2-14)	
Serum creatinine, mg/dL, median (range)	NA		0.8 (0.5-2.2)		0.8 (0.6-1.1)		0.99 (0.3-6.5)	
Urea, mg/dL, median (range)	NA		33 (17-54)		41 (31-55)		47 (20-231)	
Serum albumin, g/dL, median (range)	NA		NA		4.3 (3.5-4.8)		4.1 (2.1-9)	
Serum β2-microglobulin, mg/dL, median (range)	NA		NA		2.6 (1.1-4.3)		3.7 (1.3-52)	
Serum M-component (g/L)	NA		0.6 (0-2.2)		1.6 (0.6-3.1)		3.4 (1.6-1161)	
Heavy chain, IgA/IgG, (%)			35/65		38/62		30/70	
Light chain, κ/λ, (%)			45/55		62/38		74/26	
Number of plasma samples analyzed	13		18		7		12	

*all patients were studied at diagnosis prior to any treatment.

Copyright: © 2021 by the authors. Licensee MDPI, Basel, Switzerland. This article is an open access article distributed under the terms and conditions of the Creative Commons Attribution (CC BY) license (http://creativecommons.org/licenses/by/4.0/).
